# The zebrafish in toxicology: a bibliometric analysis reveals current trends and future avenues for predictive safety assessment

**DOI:** 10.3389/ftox.2025.1700031

**Published:** 2026-01-12

**Authors:** Carla Lima, Darlan Gusso, Geonildo Rodrigo Disner, Felipe Justiniano Pinto, Maria Alice Pimentel Falcão, João Gabriel Santos Rosa, Mônica Lopes-Ferreira

**Affiliations:** Butantan Institute, São Paulo, Brazil

**Keywords:** alternative models (3Rs), developmental stages, hazard screening, toxicological biomarkers, zebrafish toxicology

## Abstract

The zebrafish (*Danio rerio*) has become an indispensable model in toxicological research, bridging environmental monitoring, disease modeling, and preclinical drug screening. This study presents a comprehensive bibliometric and methodological analysis of 20,291 publications from 2014 to 2024, revealing distinct trends and opportunities in the field. Acute toxicity studies dominate the literature (39.36%), followed by neurotoxicity (19.50%) and immunotoxicity (11.39%), reflecting the widespread adoption of high-throughput embryonic assays such as the Fish Embryo Acute Toxicity (FET) test. While the model’s strengths in rapid hazard assessment are well-established, our analysis identifies a significant emphasis on early developmental stages (embryos and larvae), creating a critical gap in chronic toxicity evaluation and adult organism studies. Methodologically, zebrafish toxicology leverages a versatile toolkit including behavioral phenotyping, high-resolution imaging, molecular analyses, and omics technologies. However, applications often remain isolated within specific domains, highlighting the need for more integrative approaches. The field is characterized by strong growth led by China and the United States, with research published predominantly in environmental and multidisciplinary journals. Substantial numbers of studies investigating “Unclassified Compounds” indicate both innovation in studying emerging contaminants and challenges in metadata standardization. We conclude that future advancements require leveraging multi-omics integration and sophisticated transgenic tools to transform the zebrafish from a screening model into a predictive platform for systems toxicology. By addressing current limitations in life-stage representation, chronic exposure paradigms, and translational validation, zebrafish research can fully realize its potential in shaping regulatory policies and advancing personalized toxicology.

## 1 Introduction

The zebrafish (*Danio rerio*) has emerged as a preeminent model organism in biomedical and environmental research, bridging fundamental discovery and applied toxicology with remarkable efficacy. Its anatomical, genetic, and physiological conservation with mammals—coupled with external fertilization, high fecundity, and larval transparency—has positioned it as a versatile system for high-throughput screening and mechanistic investigation.

The development of the zebrafish is characterized by well-defined stages that occur rapidly and in a highly synchronized manner. After fertilization, the embryo undergoes the cleavage, blastula, gastrula, and segmentation phases, culminating in hatching around 3 dpf. During this initial phase, the organism is referred to as an embryo and still relies on the yolk as its main energy source. From hatching (3 dpf) onward, the zebrafish enters the larval stage (up to 29 dpf), which is characterized by the onset of exogenous feeding, the functional development of organs, and the establishment of major physiological pathways. Between 30 and 89 dpf in the juvenile stage, fish exhibit a fully formed body but continue to grow. Around 90 dpf, zebrafish reach sexual maturity and are considered an adult until approximately 2 years of age (Kimmel et al., 1995; Westerfield, 2000).

These attributes are reinforced by standardized international guidelines, such as those from the Organisation for Economic Co-operation and Development (OECD), which endorse protocols like the Fish Embryo Acute Toxicity (FET) test, ensuring reproducibility and regulatory relevance. Notably, ethical considerations under European Directive 2010/63/EU classify early-stage embryos (up to 120 h post-fertilization) as non-protected due to yolk-dependent nutrition and absence of independent feeding (Strähle et al., 2012; Kirk, 2018), facilitating their widespread use in compliance with the 3Rs principles (Vincent et al., 2015; Bailone et al., 2019).

Zebrafish are uniquely situated at the intersection of environmental toxicology, disease modeling, and drug discovery. They serve as sensitive biosensors for aquatic pollutants—from pesticides and industrial compounds to microplastics—and as capable models for human pathologies, including neurodegenerative, metabolic, and immune disorders. Their utility extends to preclinical drug screening, where they enable rapid efficacy and safety assessment at a fraction of the cost and time required by traditional mammalian models (Liu et al., 2014; Truong and Tanguay, 2017; Massei et al., 2023; Cao et al., 2016; Peng et al., 2021; Wang et al., 2024). This is particularly valuable given the high attrition rate of drug candidates during early development, often due to unforeseen toxicity or lack of efficacy *in vivo* (Patton et al., 2021).

In this work, we provide a comprehensive analysis of zebrafish-based toxicological and pharmacological research published between 2014 and 2024. By synthesizing data from over 20,000 studies across environmental, disease modeling, and preclinical domains, we outline the most common assays, their applications, and their limitations. We also provide a detailed guide to the core assays in zebrafish toxicology—from acute toxicity assays and high-resolution imaging to molecular and omics techniques—and discuss their application across environmental, disease modelling, and preclinical domains. This review serves as a practical guide for leveraging the strengths of the zebrafish model while also critically navigating its current constraints.

## 2 Methods

**FIGURE 1:**
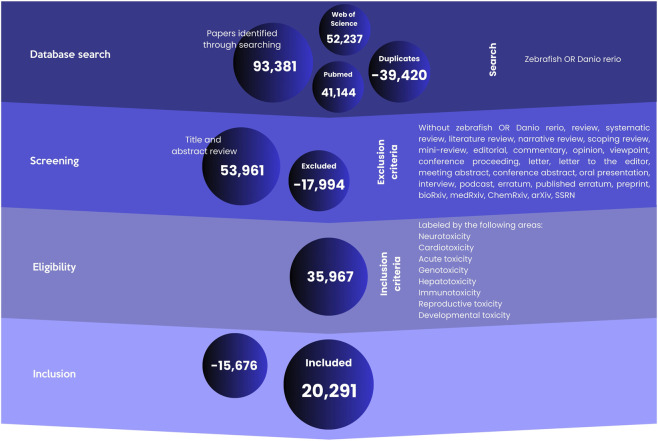
Flowchart of the search method applied to the databases. Keywords and Boolean operators were used to find articles published with the zebrafish model between January 2014 and December 2024.

A systematic literature search was conducted to identify original research articles utilizing zebrafish (*D. rerio*) as a model organism, published between 1 January 2014, and 31 December 2024. The search was performed in two major electronic databases: PubMed and Web of Science (Core Collection). The search strategy employed the key terms: “Zebrafish” OR “*D. rerio*” to maximize retrieval of relevant publications. All retrieved records were imported into Rayyan software for systematic screening and management. The initial database searches yielded a total of 93,381 records. The screening process, conducted solely based on titles and abstracts was conducted in sequential phases, as detailed in the PRISMA flow diagram (Figure 1):

1. Removal of Duplicates: 39,420 duplicate records were identified and removed, resulting in 53,961 unique articles for title and abstract screening.
2. Screening by Publication Type: Records were excluded if they were not original research articles. This included reviews (systematic, narrative, scoping, mini), editorials, commentaries, opinions, conference proceedings, letters, abstracts, preprints (e.g., from bioRxiv, medRxiv), and errata. This step excluded 17,994 records, leaving 35,967 articles.
3. Exclusion of Non-Zebrafish/Other Models: Articles were excluded if they did not primarily focus on zebrafish or if they concurrently featured other animal models (e.g., rat, mouse, murine models, rabbit, primate, canine, swine, mammalian cell lines, or human cells). This step excluded 30 records, resulting in 35,937 articles.

4. Date Filtering: To ensure strict adherence to the defined publication window, articles with an electronic publication date (ePub) of 2013 or 2025 were excluded, even if their print publication date fell within the 2014–2024 range. This final refinement resulted in a final corpus of 35,937 original research articles for inclusion in the subsequent analysis.

The final corpus of 35,937 articles was subsequently categorized according to its primary research focus on specific toxicity endpoints: Neurotoxicity, Cardiotoxicity, Acute toxicity, Genotoxicity, Hepatotoxicity, Immunotoxicity, Reproductive toxicity and Developmental toxicity (Supplementary Material 1).

Articles that did not align with these predefined toxicity domains were excluded 15,676, resulting in a refined dataset of 20,291 articles for in-depth analysis (see Supplementary Material 1). All subsequent analyses were performed on this final dataset of 20,291 publications.

Our analytical approach encompassed several key dimensions:

1. Temporal and Journal Trends: We quantified the annual publication volume and identified the top 10 journals publishing research in these toxicity domains.
2. Geographical Distribution: We determined the top 10 countries contributing to the global output in this specific field and we calculated the percentage of participation among the 10 countries that publish the most.
3. Model Application: We assessed the proportional use of zebrafish across different life stages (embryo, larva, juvenile, adult) and the adoption of *in vitro* zebrafish cell models. Information regarding the developmental stages evaluated in each study was extracted through a systematic screening of the abstract of each article, allowing the identification and classification of life-stage progression patterns.
4. Research Context Categorization: Articles were classified into broader research contexts (Environmental, Disease model, and Preclinical) to understand the primary application of the toxicity data.
5. Endpoint-Specific Analysis: Within each of the eight toxicity domains (Neurotoxicity, Cardiotoxicity, Acute toxicity, Genotoxicity, Hepatotoxicity, Immunotoxicity, Reproductive toxicity, and Developmental Toxicity) and three research contexts (Disease model, environmental and preclinical), we conducted a detailed quantitative analysis to identify the most frequently studied compounds, mechanisms, and endpoints (Supplementary Material 1).

In Supplementary Material 2, the “notes” column can be used to validate the analyses. To do so, readers should refer to the labels provided in Supplementary Material 1 and use them as a guide to interpret Supplementary Material 2, where each article is correspondingly labeled in the ‘notes’ column.

Figures 1, 3, 5 were created using Canva software, whereas Figures 2, 4, 6–8 were generated with GraphPad Prism 8.

**FIGURE 2:**
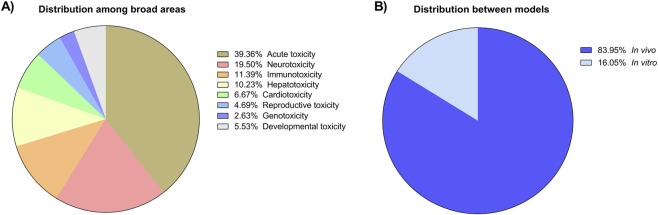
**(A)** Distribution of articles among the main toxicity areas evaluated in zebrafish, highlighting the predominance of acute toxicity studies (39.58%), followed by neurotoxicity (19.61%), immunotoxicity (11.45%), hepatotoxicity (10.29%), cardiotoxicity (6.71%), reproductive toxicity (4.72%), genotoxicity (2.65%) and Developmental Toxicity (5,56%). **(B)** Distribution of studies according to the experimental model, showing a higher proportion of *in vivo* (84%) compared to *in vitro* studies (16%).

## 3 Analysis of zebrafish publication profiles in toxicology

Our initial database search of PubMed and Web of Science yielded 93,381 records (41,144 and 52,237, respectively). Following a rigorous screening process to identify relevant toxicological studies, 20,291 articles were selected for final analysis and data extraction (Figure 1).

**FIGURE 3:**
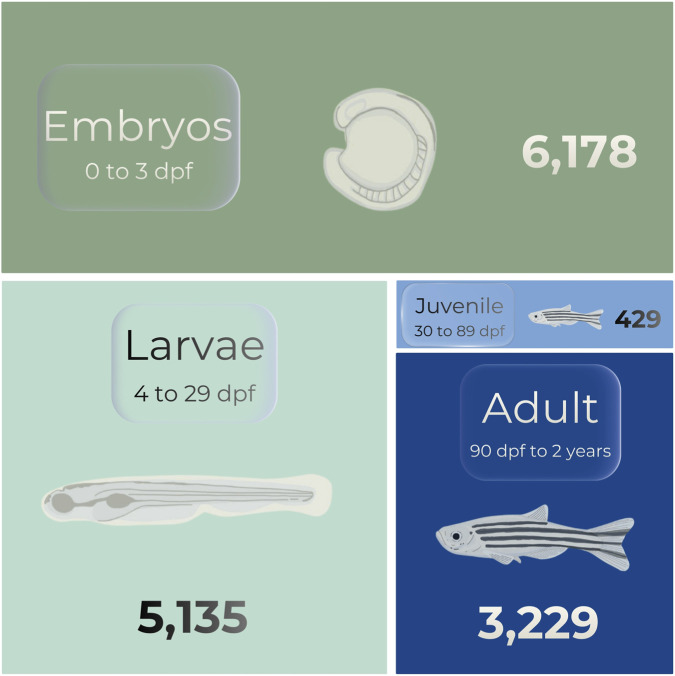
Zebrafish developmental stages used in toxicology studies. Embryos (41,3% of articles) were the most frequently employed stage, followed by larvae (34.3%), adults (21.6%), and juveniles 2.9%). The zebrafish stages are divided by embryo (0–72 hpf), larval (3–29 dpf), juvenile (30–89 dpf), and adult (≥90 dpf).

A targeted keyword analysis revealed the predominant areas of toxicological focus within zebrafish research. The most frequently represented categories were: acute toxicity studies (39.36%), followed by neurotoxicity (19.61%), immunotoxicity (11.39%), hepatotoxicity (10.23%), cardiotoxicity (6.67%), reproductive toxicity (4.69%), genotoxicity (2.63%) and Developmental Toxicity (5,53%) (Figure 2A). The vast majority of these studies (83,95%) utilized *in vivo* models, while the remaining 16,05% employed *in vitro* models (Figure 2B). Among the *in vivo* studies, embryos were the most common life stage (6,178 articles), followed by larvae (5,135), adults (3,229), and juveniles (429) (Figure 3). The analysis of developmental-stage progression revealed marked disparities in how zebrafish life stages are represented across published studies. The majority of articles followed organisms from the embryonic to the larval stage (0 hpf to 29 dpf), totaling 1,803 publications, highlighting a strong emphasis on early developmental windows. In contrast, a substantially lower number of studies extended follow-up into later stages, with only 119 articles covering the transition from the embryonic to juvenile stage (30 dpf to 90 dpf) and 691 progressing from embryonic to adult stages (3 mpf to 2 years). Transitions beginning at later stages were even less frequently represented. Specifically, 164 studies evaluated progression from the larval to juvenile stage, while 160 followed organisms from the juvenile to adult stage (Supplementary Material 1).

In addition to *in vivo* studies, a subset of research included in our analysis employed *in vitro* models, totaling 2,697 articles. These studies typically used isolated cells, organ explants, or embryo-derived tissues. Common *in vitro* systems include primary ovarian explants to study follicle growth and differentiation, cell lines such as ZF4 (zebrafish fibroblasts; 55 articles), zebrafish liver cells (33 articles), zebrafish fibroblasts (7 articles), and blastomeres (4 articles). It should be noted that because our screening was based on titles and abstracts, some included studies used *in vitro* cultured cells from other species, such as carp, to investigate zebrafish-related processes, for example as xenotransplants or for mechanistic assays indirectly related to zebrafish physiology, rather than using zebrafish cells or tissues directly.

**FIGURE 4:**
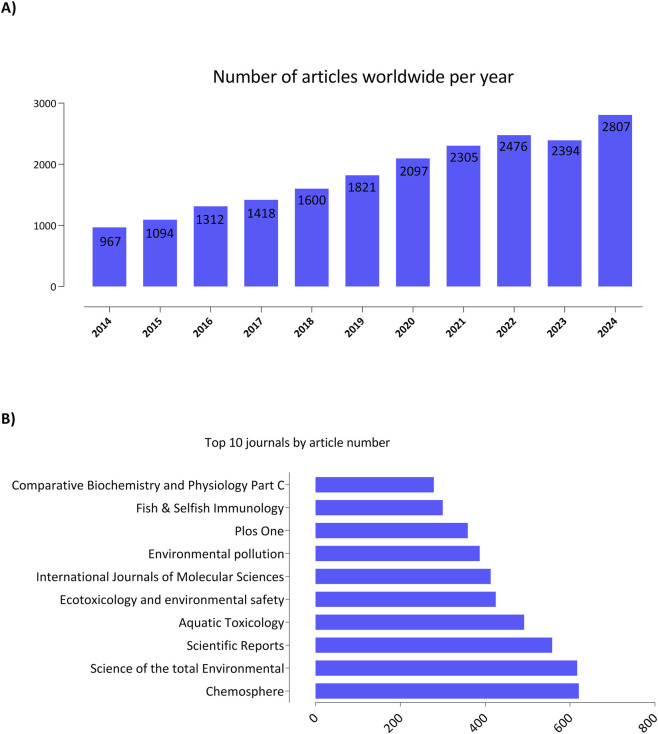
**(A)** Number of articles published annually on zebrafish in toxicology from 2014 to 2024, showing consistent growth over the years. **(B)** Ten journals with the highest number of publications, with Chemosphere, Science of the Total Environment, and Scientific Reports standing out as the main sources of dissemination. Followed by Aquatic Toxicology, Ecotoxicology and Environmental Safety, International Journals of Molecular Sciences, Environmental Pollution, PlosOne, Fish and Selfish Immunology, and Comparative Biochemistry and Physiology Part C.

**FIGURE 5:**
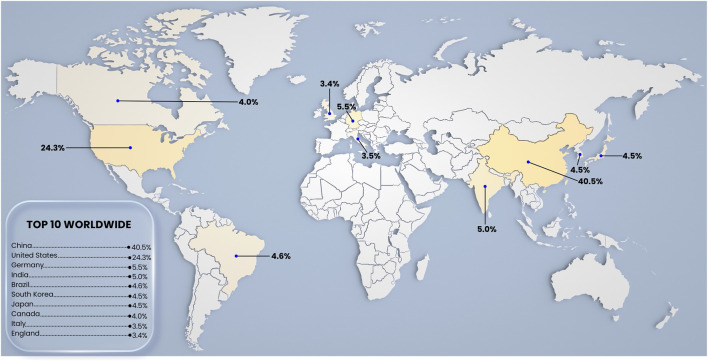
Geographic distribution of publications in toxicology using zebrafish. The map highlights the ten countries with the greatest scientific contribution, led by the China (40.5%), United States (24.34%), followed by German (5.52%), India (5.04%), Brazil (4.58%), South Korea (4.53%), Japan (4.48%), Canada (4.09%), Italy (3.47%), and England (3.46%).

Publication output demonstrated a consistent upward trend from 2014 (967 articles) to 2024 (2,807 articles), with a peak in 2022 (2,476 articles) (Figure 4A). The top 10 journals publishing this research were Chemosphere (627 articles), Science of the Total Environment (625), Scientific Reports (562), Aquatic Toxicology (492), Ecotoxicology and Environmental Safety (430), International Journal of Molecular Sciences (413), Environmental Pollution (391), PLOS One (360), Fish and Shellfish Immunology (300), and Comparative Biochemistry and Physiology Part C: Toxicology and Pharmacology (280) (Figure 4B). China was the leading country by author affiliation (5,778 articles or 40.50% of the top ten), followed by the United States (3,473 or 24.34% of the top ten), and Germany (787 or 5.5% of the top ten). India, Brazil, Japan, South Korea and Canada presented an average of 4.0% of the top ten of publications followed by Italy and England with 3.46% of the top ten (Figure 5).

**FIGURE 6:**
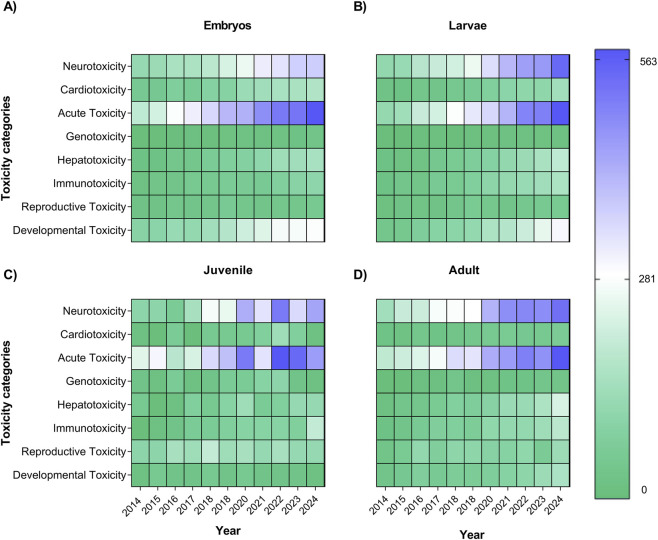
Interaction analysis between annotation dimensions. The heatmap illustrates the relationship between developmental stages (embryos - **(A)**, larvae - **(B)**, juvenile - **(C)**, and adults - (D) and toxicity categories over time. Blue shades represent higher research articles frequency in a given combination, while green indicates lower occurrence. This visualization highlights temporal trends and stage-specific focuses in zebrafish toxicology studies.

To further investigate the temporal evolution of zebrafish toxicology research, we analyzed the interaction between developmental stage and publication year (Figure 6). The heatmap reveals that studies involving embryos (Figure 6A) have progressively increased over the past decade, while investigations focusing on adults remained comparatively stable (Figure 6D). The larvae (Figure 6B) and juvenile (Figure 6C) have increased to neurotoxicity and acute toxicity since 2023. This trend highlights a growing preference for early-life exposure models, likely due to their higher experimental throughput, transparency, and relevance for developmental and mechanistic assessments.

**FIGURE 7:**
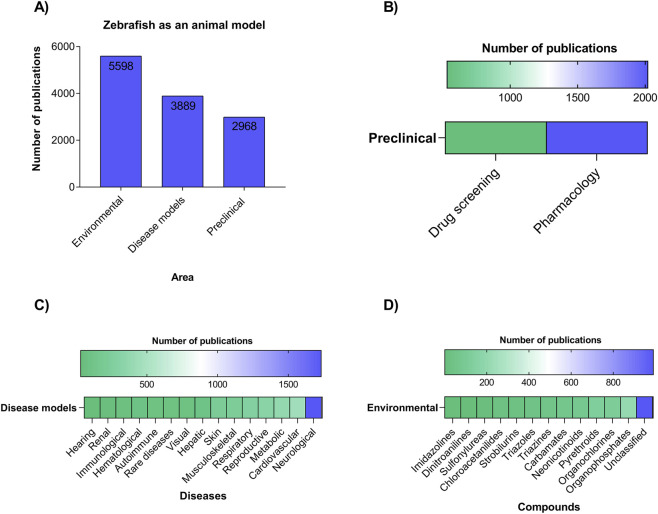
**(A)** General categories in which zebrafish are used as an experimental model, including environmental studies, disease modeling, and preclinical applications. **(B)** Classification of environmental compounds investigated, showing a predominance of organophosphates, organochlorines, and unclassified compounds. **(C)** Distribution of disease models evaluated, with an emphasis on neurological, cardiovascular, and metabolic conditions. **(D)** Preclinical applications of zebrafish, highlighting pharmacological and drug screening approaches.

Using a comprehensive keyword strategy, articles were classified into three major research domains: Environmental (5,598 articles), Disease Models (3,889 articles), and Preclinical (2,968 articles) (Figure 7A). Distribution of articles in the Preclinical category, showing a greater concentration in Pharmacology (2018 articles) when compared to Drug screening (533 articles), indicating a more significant focus on pharmacological studies in the preclinical model (Figure 7B). Distribution of articles by disease model, showing the predominance of studies in Neurological (1731 articles), followed by Cardiovascular (449), Metabolic (374), Reproductive (312) and Respiratory (276), while areas such as Musculoskeletal (231), Skin (222), Hepatic (103), Visual (76), Rare diseases (76) and Autoimmune (75) appear with less representation (Figure 7C). Distribution of articles according to pesticide classes, highlighting the predominance of Organophosphates (207) and Pyrethroids (123), followed by Organochlorines (117) and Neonicotinoids (78), while lower frequencies were observed for Carbamates (65), Triazines (51), Triazoles (43), Strobilurins (30), Chloroacetanilides (29), Sulfonylureas (13), Dinitroanilines (5) and Imidazolines (1), with a substantial number of studies classified as Unclassified (989) (Figure 7D). A significant number of articles (989) investigated Unclassified Compounds, including specific agents like glyphosate, various pesticides, herbicides, and fungicides (full list in Supplementary Material 1). It is important to note that some articles were counted in multiple categories due to overlapping research focuses.

**FIGURE 8:**
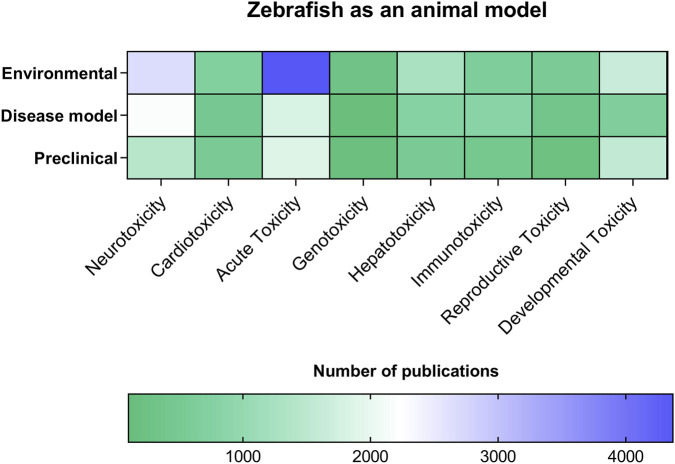
Use of Zebrafish as an Animal Model Across Different Toxicology Categories. This heatmap illustrates the distribution of scientific publications using zebrafish (*Danio rerio*) as an experimental model, organized according to Application Context (Y-axis: Environmental, Disease Model, and Preclinical) and Toxicity Category (X-axis: Neurotoxicity, Cardiotoxicity, Acute Toxicity, Genotoxicity, Hepatotoxicity, Immunotoxicity, Reproductive Toxicity, and Developmental Toxicity). Color intensity represents the total number of publications for each category combination, ranging from approximately 21 (lightest green) to approximately 4,397 (darkest blue/purple). The highest concentration of studies is observed at the intersection of the “Disease Model” context and the “Acute Toxicity” category, indicated by the darkest square on the heatmap, highlighting this area as the most extensively explored application of zebrafish in toxicological research.

The heatmap reveals a heterogeneous distribution of scientific publications employing zebrafish across different application contexts and toxicity categories. Overall, the Disease Model context demonstrated the highest concentration of studies, particularly within the Acute Toxicity category, which exhibited the greatest number of publications (approximately 4,397), representing the most intensively explored intersection in the dataset. Neurotoxicity, hepatotoxicity, and immunotoxicity also showed substantial representation, especially within the Disease Model and Preclinical contexts, indicating a strong focus on using zebrafish to investigate systemic and organ-specific toxicological effects. In contrast, developmental and genotoxic toxicity categories displayed comparatively lower publication frequencies across all application contexts, with the lowest values observed in specific combinations reaching approximately 21 publications. The Environmental context exhibited a more uniform but generally lower distribution of studies across toxicity categories when compared to Disease Model and Preclinical applications (Figure 8). These results highlight a clear research trend toward leveraging zebrafish primarily for disease-related toxicological investigations, particularly acute toxicity, while certain toxicity domains remain comparatively underexplored.

## 4 Analysis methods applied to different areas of research

Although manuscripts were categorized into distinct domains, the experimental methodologies employed (e.g., behavioral assays, genomic analysis, physiological endpoints) are highly convergent and often applied across environmental, disease modeling, and preclinical contexts. This methodological synergy allows zebrafish to serve as a versatile model for translational research.

To illustrate this unified approach, the following section details the core toxicological and functional assays prevalent in zebrafish research. This description serves as a guideline for employing zebrafish in toxicology, outlining the principles, applications, and relevance of key assays for evaluating a wide spectrum of biological processes and toxicological endpoints.

### 4.1 The fish embryo acute toxicity (FET) test

The Fish Embryo Acute Toxicity (FET) test, formalized as OECD Test Guideline 236 in 2013, provides a well-established alternative to the conventional acute toxicity test using juvenile or adult fish (OECD 203) (Xu et al., 2023; OECD, 2013). Its principle involves exposing freshly fertilized zebrafish eggs (within 3 h post-fertilization, hpf) to a test compound for a period of 96 h. Lethality and apical morphological endpoints are assessed at 24-h intervals. The key validated endpoints include: (1) coagulation of fertilized eggs, (2) lack of somite formation, (3) failure of tail-bud to detach from the yolk sac, and (4) absence of a heartbeat. A defining feature of this protocol is that the test medium is not renewed, and deceased larvae are not removed during the exposure period, with cumulative mortality quantified at the 96-h endpoint.

For a reliable execution of the FET test, several critical drug-specific properties must be considered. The compound must exhibit sufficient aqueous solubility and appropriate lipophilicity to ensure bioavailability in the aqueous exposure medium. The use of solvents, such as DMSO, is permissible but should not exceed a concentration of 0.01% (v/v) to avoid confounding embryotoxicity (Hoyberghs et al., 2021). Consequently, polar or charged drugs that exhibit negligible passive diffusion across biological membranes are generally poor candidates for this assay. In contrast, low-molecular-weight ligands and peptides, which demonstrate more favorable penetration kinetics, are well-suited for this model (Rautio et al., 2018). Finally, the inclusion of a positive control is essential to validate the responsiveness of each experimental batch. This feasible and standardized approach has significantly contributed to the toxicological knowledgebase, proving highly valuable for the early generation of drug safety alerts.

The FET test provides visualization of *in vivo* effects in an alternative translational model with a finely regulated response to that of mammals (Ball et al., 2025). The use of alternative models such as zebrafish helps to understand and map toxic effects of constantly used compounds and to screen compounds in development (Johnson et al., 2020).

### 4.2 Neurotoxicity

Neurotoxicity encompasses the adverse structural or functional damage to the nervous system resulting from the direct or off-target effects of chemical compounds. These alterations can manifest as a spectrum of temporary or permanent deficits, ranging from subtle behavioral changes to severe neurological disorders. The zebrafish has emerged as a powerful model for neurotoxicity screening due to its high degree of evolutionary conservation in key neurobiological pathways. The larval zebrafish brain possesses neuromodulatory circuits homologous to those in mammals, including major neurotransmitter systems such as glutamate, GABA, acetylcholine, dopamine, serotonin (5-HT), noradrenaline, and histamine (Rosa et al., 2022). The above-mentioned neurotransmitters are very similar to those found in mammals (including humans). The evolutionary conservation of these molecules suggests that the basic mechanisms of neural communication were established very early in the history of life, being essential for the functioning of the nervous system in various animal phyla, including mammals. Not only are the signaling and response similar, but so is the neuroanatomy (Howe et al., 2013; Stewart et al., 2014).

Complementing these neurogenetic similarities, zebrafish exhibit a rich and quantifiable repertoire of behaviors in response to environmental and pharmacological stimuli. These include spontaneous movements, stimulation-induced escape responses, and complex swimming patterns (D’Amora and Giordani, 2018; Zhang et al., 2018; Wu et al., 2021). This behavioral complexity enables the development of robust screening approaches. A primary method involves the analysis of locomotor activity, often assessed through parameters like total distance travelled and mean velocity. These studies are frequently complemented with light-dark transition paradigms, a well-established assay for detecting neurobehavioral perturbations (Koseki et al., 2014).

The normal locomotor pattern of zebrafish larvae is characterized by a distinct phenotype: immediate and robust hyperactivity during dark periods followed by significantly lower activity during light periods. Deviations from this pattern are indicative of neurotoxic or psychoactive effects. Furthermore, higher-order behaviors such as anxiety-like responses can be quantified in older larvae (e.g., 5 days post-fertilization) by measuring thigmotaxis—the propensity to remain near the edges of an arena, which is a conserved vertebrate behavior analogous to rodent open-field test behavior (Han et al., 2021). The quantification of these endpoints is facilitated by high-throughput automated tracking systems (e.g., ZebraBox™, DanioVision™), allowing for the efficient and objective screening of compounds for potential neurotoxicity.

### 4.3 Hepatotoxicity

The zebrafish has emerged as a highly relevant model for assessing drug-induced liver injury (DILI) due to the conserved functional and developmental characteristics of its hepatic system. Liver organogenesis in zebrafish begins around 28 h post-fertilization (hpf) and is largely complete by 72 hpf, resulting in a fully functional organ capable of vital processes such as bile secretion (Menke et al., 2011). This bile, predominantly composed of bile alcohols, is essential for both the excretion of toxic substances and the digestion of dietary lipids (Ellis et al., 2018). Crucially, the zebrafish liver possesses sophisticated xenobiotic metabolism machinery, including both Phase I and Phase II biotransformation enzymes, enabling a response to chemical insults that is translationally relevant to mammals (Alderton et al., 2010).

Leveraging this physiological conservation, several robust assays have been developed to evaluate hepatotoxicity in larval zebrafish. A common endpoint involves the assessment of overall liver function by analyzing bile secretion and lipid metabolism. Impaired liver function disrupts the metabolism and absorption of yolk lipids, leading to their abnormal retention. This can be visualized and quantified histologically using Oil Red O staining to mark neutral lipids. For more specific and sensitive detection of hepatic steatosis, fluorescent probes such as BODIPY-labeled fatty acids can be employed to label accumulated lipids *in vivo* (Balamurugan et al., 2022).

Functional digestive capacity can be assayed using the reporter molecule PED-6, a quenched fluorescent substrate that becomes strongly fluorescent upon cleavage by bile salt-activated lipases in the intestinal lumen, providing a direct readout of biliary function (Bellver et al., 2021). Beyond these morphological and functional endpoints, molecular biomarkers offer enhanced sensitivity. Notably, the liver-specific microRNA, miRNA-122, has been validated as a highly sensitive and conserved biomarker for hepatotoxicity in both human studies and zebrafish models, providing a quantifiable and early indicator of hepatocellular damage (Vliegenthart et al., 2014). Zebrafish model allows for rapid toxicity assessment in a whole vertebrate organism and evaluates compounds that assess hepatotoxicity in mammals, such as Classic Liver Injury disruptions: The assessment of liver enzymes released into plasma in case of cell damage, such as Aspartate Aminotransferase (AST), Alanine Aminotransferase (ALT), and Alkaline Phosphatase (ALP), provides a direct and conserved reading of hepatocellular injury (Martins et al., 2021). This combination of phenotypic and molecular endpoints makes the zebrafish larvae a powerful platform for high-throughput hepatotoxicity screening.

### 4.4 Cardiotoxicity

Drug-induced cardiotoxicity remains a leading cause of drug attrition during clinical trials and post-market withdrawals, underscoring the critical need for its early detection in preclinical safety assessment (Varga et al., 2015). The zebrafish model has proven invaluable for this purpose, offering unparalleled capabilities for the direct visualization of cardiac structures and the functional analysis of hemodynamics *in vivo*. The high level of genetic and physiological conservation with the human heart makes it a translationally relevant model for predicting cardiac effects in mammals (Alzualde et al., 2018).

The zebrafish heart, comprising a single atrium and ventricle, shares fundamental structural and functional conservation with mammals. Each chamber is lined by an endocardial layer and surrounded by myocardium, and these tissues can be visualized with cellular resolution using standard microscopy, enabling clear distinction between normal and pathological phenotypes. Cardiac function begins early, with the heart tube initiating contractions at approximately 24 h post-fertilization (hpf) at a rate of 40–50 beats per minute (bpm). Circulation commences shortly thereafter, and the heart rate rapidly increases to a baseline of 120–180 bpm (De Luca et al., 2014; Yalcin et al., 2017). Remarkably, despite the embryo’s minute size, techniques have been established to obtain electrocardiogram (ECG) readings from 3 days post-fertilization (dpf) onwards, revealing waveforms and conduction properties analogous to those of adult zebrafish and other vertebrates (Dhillon et al., 2013).

Cardiotoxic insults in zebrafish larvae manifest through a range of quantifiable morphological and functional endpoints. Impaired cardiac development or function is frequently characterized by pericardial edema, reduced blood flow, chamber congestion, and arrhythmias stemming from contractility or conduction abnormalities. Comprehensive analysis typically involves assessing both morphology (e.g., pericardial area, thrombosis, hemorrhage) and key hemodynamic parameters. Critical functional metrics include heart rate, stroke volume, cardiac output, fractional shortening (a measure of contractility), and vascular flow velocities (Narumanchi et al., 2021; Sampurna et al., 2019). While initial screening can be performed using inverted or stereo brightfield microscopy (Houk and Yelon, 2016), the adoption of automated systems (e.g., ZebraBox, Noldus DanioVision) and specialized software (e.g., Zcardio®) now enables high-throughput, accurate quantification of these parameters from high-speed video recordings, significantly enhancing the precision and efficiency of cardiotoxicity screening.

### 4.5 Reproductive toxicity

Although zebrafish is not an ideal model for understanding mammalian reproduction because since it lacks dimorphic sex chromosomes and the male heterogametic system, as well as the SRY gene that triggers male differentiation in mammals (Piprek, 2010), its remains a powerful model to evaluate the effects of environmental or pharmacological compounds on reproductive hormones, gonadal development, and gametogenesis. The zebrafish model offers significant translational value for reproductive toxicity testing due to its conserved endocrine physiology. Throughout its life cycle, the zebrafish produces key reproductive hormones homologous to those in humans, including gonadotropin-releasing hormone (GnRH), follicle-stimulating hormone (FSH), luteinizing hormone (LH), and the sex steroids testosterone, estrogen, and progesterone (Hara et al., 2016). The onset of endocrine activity follows a defined developmental timeline: GnRH is detectable within the first 10 days post-fertilization (dpf), followed by the appearance of FSH, LH, and steroid hormones during the juvenile stage (15–30 dpf). Sexual maturation concludes around 30 dpf with the complete formation of gonads. A critical marker of reproductive maturity in females is the production of vitellogenin, a yolk precursor glycoprotein synthesized in the liver under estrogenic stimulation, released into the bloodstream, and incorporated into developing oocytes via receptor-mediated uptake—a process highly conserved across vertebrates (OECD, 2011; He et al., 2014).

Standardized protocols have been established to evaluate the effects of chemical exposure on reproductive fitness. These assays typically employ wild-type zebrafish (7–8 months old) exposed to at least three concentrations of a test compound. To establish a robust baseline, fish of uniform size are pre-screened for successful spawning over a two-week period. For testing, treated females and males are fed normally and then placed overnight in separate spawning tanks, paired with unexposed, negative control siblings to minimize mate preference variability. The following morning, dividers are removed at the onset of light, and fish are allowed to spawn for 1 hour. Successful fertilization is confirmed by the presence of cleaving eggs. Fecundity is quantified by counting the total number of eggs oviposited per female. To ensure statistical reliability, this entire procedure is repeated across three independent trials using distinct groups of fish.

Beyond fecundity, the assay can be extended to evaluate behavioral endpoints. Male reproductive behavior—a sensitive indicator of endocrine disruption—is assessed by recording interactions for a defined period (e.g., 10 min). Key courtship behaviors, such as the frequency of male approaches and initiation of mating rituals, are quantified for each tank (Marvel et al., 2021). This integrated approach, combining physiological output with behavioral analysis, provides a comprehensive assessment of compound effects on reproductive health.

### 4.6 Genotoxicity

The comet assay (single-cell gel electrophoresis) is a highly sensitive and versatile method for detecting DNA strand breaks at the individual cell level, making it a cornerstone of genotoxicity assessment. Its primary advantage lies in its broad applicability to any nucleated cell type that can be dispersed into a viable single-cell suspension. In zebrafish research, this assay is routinely performed on nucleated erythrocytes (a readily accessible cell type) as well as on whole embryos, enabling comprehensive evaluation of genetic damage across different biological matrices (Gülsoy and Mutlu, 2015; Ku-Centurión et al., 2016; D’Costa et al., 2018). For solid tissues, effective cell isolation is achieved through methods such as mechanical homogenization, mincing, enzymatic digestion, or mesh filtration. To ensure the integrity of results, it is critical to protect cell suspensions from exposure to light and elevated temperatures throughout the preparation process to avoid inducing artifactual DNA damage. A minimum cell viability of 70% is recommended prior to assay initiation to confirm sample quality.

The technical execution of the comet assay involves a series of standardized steps. First, the prepared cell suspension is embedded in a thin layer of agarose on a microscope slide to immobilize the cells. Subsequent lysis in a high-salt, detergent-based buffer removes cellular and nuclear membranes, histones, and other proteins, liberating the DNA while maintaining its higher-order structure (nucleoid). Following lysis, slides are immersed in an alkaline electrophoresis buffer to allow DNA unwinding and the expression of alkali-labile sites as strand breaks. During electrophoresis, fragmented DNA and relaxed loops migrate away from the intact, supercoiled DNA that remains in the nucleoid core. This migration creates the characteristic “comet” appearance, with the head representing intact DNA and the tail consisting of damaged fragments.

The analysis and quantification of DNA damage are performed according to internationally validated guidelines (OECD, 2016). The extent of genotoxicity is quantitatively assessed by measuring parameters such as tail intensity (% DNA in tail), tail moment, or tail length. This scoring can be conducted using manual, semi-automated, or fully automated image analysis systems, ensuring objective and reproducible quantification of DNA damage for robust safety assessment.

The zebrafish represents a valuable translational model for genotoxicity assessment, as many of its DNA repair pathways and cellular responses to genetic damage are conserved in mammals. The presence of homologous genes involved in base excision repair, nucleotide excision repair, and double-strand break repair allows zebrafish assays to provide relevant insights into the mechanisms of DNA damage and mutagenesis observed in higher vertebrates (Canedo and Rocha, 2021). Moreover, its transparent embryos and rapid development enable real-time visualization of genotoxic effects, facilitating the identification of compounds with potential genotoxic risks in mammals.

### 4.7 *In vivo* imaging and histopathological analysis

The zebrafish model is unparalleled in its capacity for high-resolution *in vivo* imaging, a feature central to its utility in both developmental biology and toxicological research. The natural transparency of embryos and early larvae permits direct, non-invasive observation of morphogenesis, tissue formation, and regenerative processes in real time. This visualization is facilitated by mounting multiple embryos in agarose molds (whose refractive index closely matches water) for examination under stereo microscopes equipped with brightfield and darkfield illumination. For dynamic processes like epiboly, time-lapse recording provides critical insights, while computational extended depth-of-focus projections from widefield Z-stacks enable detailed analysis across numerous samples simultaneously (Bruce, 2015).

Beyond live imaging, classical histological techniques remain foundational for elucidating biological and pathological changes at the cellular and tissue levels. Paraffin embedding and cryosectioning are standard methods for preparing zebrafish tissues (Stutts et al., 2020). Staining protocols, including hematoxylin and eosin (H&E) for general morphology, Alcian blue for cartilage, and Oil Red O for lipids (as applied in hepatotoxicity assessments; Balamurugan et al., 2022) are routinely employed to evaluate organ-specific alterations. In toxicology, these methods are indispensable for identifying compound-induced damage, such as cellular degeneration, necrosis, or fibrosis in organs like the liver and kidney (Dai et al., 2014).

Molecular localization is achieved through immunohistochemistry (IHC) and immunofluorescence (IF), leveraging antibody-antigen interactions. While direct methods use a primary antibody conjugated to a fluorophore (Janardhan et al., 2018), the indirect method—particularly via biotin-avidin amplification—is most common in zebrafish due to its enhanced sensitivity (Köktürk and Altındağ, 2024; Sukswai and Khoury, 2019). This allows precise spatial mapping of biomarkers, such as those identified in earlier sections (e.g., miRNA-122 for hepatotoxicity, or specific neurotransmitters in neurotoxicity).

The true power of the zebrafish system emerges from its integration with genetic and optical technologies. Numerous transgenic fluorescent lines enable live, spatial-temporal imaging of biological processes—from cardiovascular function to neuronal activity—with subcellular resolution using confocal microscopy. This permits quantitative 4D tracking (x, y, z, time) of dynamic events over milliseconds to days (Icha et al., 2016), aligning with functional endpoints described in prior sections (e.g., locomotor behavior, cardiac output).

In conclusion, zebrafish provide a comprehensive phenotyping toolkit that synergizes *in vivo* imaging, histopathology, and molecular staining. This integrated approach is highly effective for predicting adverse effects, whether hepatic, cardiac, neurological, or reproductive, enabling more informed candidate selection and safer design of clinical studies.

### 4.8 Complementary molecular and biochemical assays

Beyond the phenotypic and histopathological endpoints detailed in previous sections, a comprehensive understanding of toxicological mechanisms requires molecular and biochemical profiling. Recent methodological advances have greatly expanded the toolkit available for zebrafish research, enabling precise detection of mRNA, proteins, and enzymatic activities relevant to toxicological responses. These include well-established techniques such as colorimetric and fluorometric assays, Western blot (WB), enzyme-linked immunosorbent assay (ELISA), high-performance liquid chromatography (HPLC), mass spectrometry, and a suite of genetic methods.

A significant challenge in zebrafish proteomics is the limited availability of species-specific antibodies. While the use of such antibodies yields highly sensitive results for detecting proteins induced by drug exposure, anti-human or anti-mouse antibodies often fail due to lack of cross-reactivity with zebrafish epitopes. Where antibodies are available, WB analysis can be performed on all life stages, typically requiring pools of at least 50 embryos or larvae to obtain sufficient protein.

To overcome these limitations, gene expression analysis has become a cornerstone for elucidating systemic toxicological mechanisms. Transcriptomic approaches allow researchers to investigate molecular responses such as drug metabolism, endocrine disruption, oxidative stress, and immune modulation, even at low exposure doses. The range of techniques spans from targeted analysis of individual genes using real-time quantitative polymerase chain reaction (rt-qPCR) or whole-mount *in situ* hybridization (WISH), the latter being exceptionally valuable in transparent embryos for spatial resolution of organ-specific gene expression (Thisse and Thisse, 2008; Vauti et al., 2020), to untargeted genomic-scale profiling.

Microarrays and, more powerfully, RNA sequencing (RNA-seq) provide genome-wide coverage of transcriptomic changes. RNA-seq offers superior sensitivity for detecting low-abundance transcripts, novel isoforms, and nuanced expression patterns, a capability greatly enhanced by the completion of the zebrafish genome sequence (Howe et al., 2013). Emerging methodologies like RNAscope™ enable multiplexed detection of up to three different RNA targets in whole-mount embryos with subcellular resolution, allowing for sophisticated RNA-protein co-localization studies (Gross-Thebing, 2020). Furthermore, single-cell RNA sequencing (scRNA-seq) reveals cellular heterogeneity and dynamic responses within tissues, offering unprecedented resolution in mechanistic toxicology. Integration of transcriptomic data with other omics layers (genomic, epigenomic, proteomic) promises a more holistic understanding of chemical-induced disruptions (Hu et al., 2023; Siddiqui et al., 2023).

Sample preparation for these assays is flexible across development: whole embryos or larvae are commonly used, with tissue-specific sampling feasible in juveniles and adults. These molecular tools are extensively applied to quantify biomarkers of endocrine and reproductive disruption, including hormones such as testosterone, estrogen, progesterone, FSH, LH, T3, T4, and GnRH, as well as neurotransmitters (e.g., dopamine, serotonin) and stress hormones like cortisol via ELISA. Additionally, colorimetric and fluorometric assays are widely employed to measure oxidative stress parameters, including glutathione (GSH), reactive oxygen species (using DCF assay), and antioxidant enzymes (SOD, CAT, GPx) (Alderton et al., 2010; Chen et al., 2018; Audira et al., 2021).

When combined with the phenotypic, behavioral, and imaging endpoints described throughout this review (such as hepatotoxicity screening using lipid staining, neurobehavioral assessment, cardiac function analysis, and reproductive output), these molecular assays form a multilevel analytical framework. This integration allows researchers to correlate molecular initiating events with functional outcomes, bridging the gap between environmental toxicology, disease modeling, and preclinical research. The zebrafish thus emerges as a deeply integrative model, capable of supporting high-throughput systems toxicology approaches that are critical for modern safety assessment and translational science.

## 5 Discussion

Our analysis of the current landscape of zebrafish toxicological research reveals a rapidly expanding field characterized by distinct methodological and thematic trends. The pronounced dominance of acute toxicity studies, representing 39.36% of all research, underscores the zebrafish’s established role in rapid initial hazard screening. This emphasis is largely driven by regulatory requirements and the widespread adoption of the Fish Embryo Toxicity (FET) test, which offers substantial advantages in cost, throughput, and ethical compliance, as embryos up to 120 hpf are not classified as protected vertebrates under many regulations (Author Anonymous, 2010; Hoyberghs et al., 2024). However, this focus raises important questions about potential gaps in chronic toxicity assessment, which is often more relevant for comprehensive human and environmental risk evaluation.

The high representation of neurotoxicity studies (19.5%) reflects the sensitivity of behavioral assays as integrative endpoints for acute toxicity assessment. Many compounds inducing acute effects also impair locomotor activity, exploratory behavior, or sensorimotor responses, making behavioral tests valuable proxies for neurotoxic outcomes (Park et al., 2021; Nabinger et al., 2021). Similarly, the significant number of immunotoxicity studies (11.39%) aligns with the developmental timeline of the zebrafish immune system, where innate immunity dominates until approximately 4 weeks post-fertilization, followed by the maturation of adaptive defenses (Cornet et al., 2020).

The clear preference for *in vivo* models (84% of studies), particularly embryonic and larval stages, highlights the practical advantages of these early developmental phases. Embryos (0–72 hpf) offer transparency, external development, and suitability for high-throughput screening, while larvae (3-7 dpf) provide functional complexity with active sensory systems and locomotor behavior while maintaining optical accessibility (Kelly and Benson, 2020; Yumnamcha et al., 2024). However, this emphasis creates a significant gap in understanding toxic effects in fully developed organisms, where organ complexity, metabolic maturity, and immune competence more closely resemble mammalian systems. This developmental bias is further evidenced by the limited progression of studies across later life stages. While a substantial number of publications followed organisms from the embryonic to larval stage, transitions into juvenile and adult phases were markedly less frequent. Such discontinuity in developmental follow-up suggests that potential delayed, cumulative, or life-stage-specific toxic effects remain underexplored. Notably, many studies and laboratories adopt the adult zebrafish exclusively, particularly in disease modeling and environmental toxicology, without integrating earlier developmental stages into their experimental design.

The exponential increase in publication output, led by China and the United States (together responsible for 64.84% of global research among the top ten countries by publication volume), reflects significant national investment and the expanding adoption of this model across diverse research communities. This growth has been accelerated by several factors: the implementation of 3Rs principles reducing mammalian use (Grimm, 2024), regulatory flexibility for embryos and larvae up to 5 dpf, and the advent of accessible genetic tools like CRISPR/Cas9 (Hwang et al., 2013). The profile of leading journals, predominantly in environmental science and multidisciplinary fields, illustrates the dual nature of zebrafish applications—serving as a fundamental pillar in aquatic ecotoxicology while gaining significant traction in biomedical research.

The substantial number of studies investigating “Unclassified Compounds” suggests the field is actively researching emerging contaminants while also potentially indicating inconsistencies in keyword standardization that may challenge future meta-analyses. The assignment of articles to multiple categories reflects the model’s remarkable versatility, where a single study might investigate pesticide neurotoxicity while bridging environmental and disease model domains through preclinical screening approaches.

Beyond publication trends, the methodological approaches themselves warrant critical evaluation. While zebrafish offer an exceptional toolkit—from high-throughput behavioral phenotyping and molecular analyses to advanced imaging—their application often remains isolated within specific toxicological domains. The heavy reliance on embryonic stages, though methodologically convenient, limits translation to later life stages where biological complexity better approximates human physiology. Moreover, the predominance of acute exposure scenarios overlooks chronic, low-dose exposures more relevant to human environmental health risk assessment. In this context, the heatmap analysis further reinforces a research bias toward disease-oriented acute toxicity studies, particularly within the Disease Model application context. While this focus highlights the utility of zebrafish for rapid toxicological screening and mechanistic insights into immediate pathological responses, it also reveals a discrepancy between experimental design and real-world exposure conditions, which are typically characterized by prolonged and low-intensity contact with environmental contaminants.

The comparatively lower representation of studies addressing developmental, genotoxicity, and environmental toxicity suggests that critical aspects of long-term biological impact remain insufficiently explored. Given the sensitivity of zebrafish to subtle developmental and molecular perturbations, expanding research efforts toward chronic exposure models and environmentally realistic scenarios could significantly enhance its relevance for predictive toxicology and risk assessment frameworks. Such a shift would allow for a more comprehensive understanding of cumulative and transgenerational effects, ultimately strengthening the translational value of zebrafish-based toxicological research.

Future directions should focus on leveraging the full potential of zebrafish methodologies through integrative approaches. The combination of high-content imaging with multi-omics technologies offers pathways to uncover novel toxicity mechanisms and biomarkers. Developing more sophisticated transgenic reporter lines will enable real-time tracking of physiological responses, while machine learning tools can enhance objectivity in behavioral and morphological analysis. Furthermore, the growing opportunity to use zebrafish for quantitative risk assessment and personalized medicine—by combining human-relevant *in vitro* models with zebrafish *in vivo* validation—promises to bridge the gap between embryonic and adult toxicology.

Despite the zebrafish’s high genetic and physiological similarity to humans, important differences in metabolism and biological processes remain (Howe et al., 2013). These differences do not disqualify the model but emphasize the need for complementary approaches using multiple species, as recommended by toxicological screening guidelines. By addressing these challenges and embracing advanced methodologies, the zebrafish model can solidify its role as a comprehensive and predictive platform for 21st-century toxicology, capable of spanning environmental monitoring, disease modeling, and preclinical assessment while providing insights that are both mechanistically profound and translationally relevant.

## 6 Conclusion

In conclusion, this analysis confirms the zebrafish as an indispensable model in modern toxicology, characterized by dynamic growth and methodological sophistication. Its established role in high-throughput acute toxicity screening—driven by regulatory adoption, cost efficiency and ethical advantages of embryonic stages—remains a cornerstone of its utility, particularly in environmental risk assessment. However, the current emphasis on acute exposures and early developmental stages reveals a critical gap in chronic and life-stage-specific toxicological evaluation, limiting comprehensive risk assessment relevant to human health.

The model’s expanding application in neurotoxicity, immunotoxicity, and biomedical research reflects its versatility and capacity to bridge mechanistic discovery and translational applications. Nevertheless, the geographic concentration of research output and inconsistencies in compound classification highlight opportunities for greater standardization, international collaboration, and harmonization of reporting practices.

Looking forward, the integration of emerging technologies—such as multi-omics, machine learning, and advanced transgenic tools—offers a pathway to transcend current limitations. By coupling high-throughput capabilities with sophisticated chronic exposure models and molecular profiling, the zebrafish can evolve from a screening tool to a systems-level platform for predictive toxicology. To fully realize these potential, future efforts could include later life stages, endocrine and epigenetic endpoints, and cross-species validation approaches. Through such strategies, the zebrafish will not only address existing gaps in toxicity testing but also accelerate the development of safer compounds and more precise environmental health policies.
