# Supplementary material for: The zebrafish in toxicology: a bibliometric analysis reveals current trends and future avenues for predictive safety assessment
Source: Front Toxicol. 2026 Jan 12;7:1700031. doi: 10.3389/ftox.2025.1700031 (PMC12832328; doi:10.3389/ftox.2025.1700031)
Supplement: Supplementary file 1 [file Supplementaryfile1.pdf]

Data to generate figure 1:

Search with the following **Keywords** Zebrafish OR Danio rerio

PubMed = 41,144

Web of Science = 52,237

Total = 93,381

Duplicates removed:

Remaining = 53,961

1st Exclusion:

"review, systematic review, literature review, narrative review, scoping review, mini-review, editorial, commentary, opinion, viewpoint, conference proceeding, letter, letter to the editor, meeting abstract, conference abstract, oral presentation, interview, podcast, erratum, published erratum, preprint, bioRxiv, medRxiv, ChemRxiv, arXiv, SSRN"

Total excluded: 6,635

Remaining: 47,326

1st Inclusion:

Keywords "zebrafish, Danio rerio OR zebra fish"

Total included: 35,967

11,359 did not mention zebrafish or Danio rerio in title or abstract and were excluded.

2nd Exclusion:

Keywords rat, rats, mouse, mice, murine, rodent, hamster, guinea pig, rabbit, monkey, macaque, primate, canine, dog, swine, pig, porcine, sheep, goat, bovine, cow, cat, cell line, human cell. As well, articles accepted in 2013 and those who were published in 2025.

Total excluded (zebrafish mentioned + other models): 10,465

Data to generate figure 2:

2nd Inclusion: Next step with 35,662 articles: Split by study area

1st Inclusion to Neurotoxicity **Keywords**

neurotoxicity, neurobehavioral, neuronal damage, dopaminergic, acetylcholinesterase, brain oxidative stress, neurotransmitter, behavior, neurobehavioural, neurotoxic, neurodevelopment, neuroinflammation, neurodegeneration, CNS, neural toxicity, neuroinflammatory, neuronal toxicity

Articles found: 5,182 (19,50%)

1st Inclusion to Cardiotoxicity **Keywords**

cardiotoxicity, heart rate, bradycardia, pericardial edema, circulation, arrhythmia, cardiac output, heart beats

Articles found: 1,772 (6,67%)

1st Inclusion to Acute toxicity **Keywords**

acute toxicity, LC50, mortality, lethality, survival, exposure, acute exposure, OECD 203, OECD 236, FET

Articles found: 10,459 (39,36%)

1st Inclusion to Genotoxicity **Keywords**

genotoxicity, DNA damage, comet assay, micronucleus, oxidative DNA damage, chromosome aberration

Articles found: 699 (2,63%)

1st Inclusion to Hepatotoxicity **Keywords**

hepatotoxicity, liver damage, steatosis, hepatocyte vacuolization, oxidative stress, liver enzyme

Articles found: 2,718 (10,23%)

1st Inclusion to Immunotoxicity **Keywords**

immunotoxicity, inflammation, cytokine, interleukin, innate immunity, macrophage, neutrophil, immune suppression, immunity

Articles found: 3,027 (11,39%)

1st Inclusion to Reproductive toxicity **Keywords**

reproductive toxicity, fertility, spawning, fecundity, ovary, testis, sex hormones, endocrine disruption, steroids

Articles found: 1,246 (4,69%)

1st Inclusion to Developmental toxicity **Keywords**

developmental toxicity, developmental toxicology, embryo toxicity, embryotoxicity, embryonic toxicity, larval toxicity, early-life stage toxicity, early developmental exposure, toxic effects on development, sublethal developmental effects

Articles found: 1,469 (5,53%)

Relative proportions: Using the keywords described above for each of the areas, we labeled 26,576 articles considering the overlaps.

Total labeled articles: 20,291

Total of labels (due to overlap): 25,102

Articles not matching any keyword: 15,371 (excluded)

Here, we arrived at the number of 20,291 articles to follow the next steps such as the division by specific areas and key words inside each area:

1st Evaluation by specific areas:

1st Evaluation by specific areas - Keywords to Neurotoxicity.

Light/Dark preference, Light/Dark test, Novel Tank Test, Locomotor activity, Thigmotaxis, swimming, Visual motor response, Startle response, escape response, Optokinetic response, Anxiety-like behavior, Seizure-like behavior, Optomotor, aversive, Shoaling, social behavior, Aggressive, Acetylcholinesterase (AChE), Dopaminergic neuron, dopamine, Neurotransmitter, GABA, serotonin, Brain oxidative stress, MDA, SOD, CAT, cortisol, Apoptosis, Caspase-3, TUNEL, caspase-8, GFAP expression, Myelin staining

Total: 7,099

1st Evaluation by specific areas - Keywords to Cardiotoxicity

Heart rate measurement (bpm), Pericardial area/edema analysis, blood flow, Circulatory defects, Atrium, ventricle, size and contraction, Arrhythmia quantification, Heart morphology, Blood pooling

Total: 560

1st Evaluation by specific areas - Keywords to Acute toxicity

Lethality rate, Survival curves, Developmental delay, Coagulation, Hatching delay, Morphological malformations, Loss of Equilibrium (LOE), Immobility, Sedation, Body decoloration, Tail abnormality, Gill dysfunction, Oedema, Hemorrhage, Respiratory Rate, Sublethal effects, Test concentration, Endpoint concentration, NOEC, LOEC, Observation time, Acute Necrosis, pigmentation absence, pigmentation loss

Total: 777

1st Evaluation by specific areas - Keywords to Genotoxicity

Micronucleus test, Chromosome aberration, γH2AX staining, DNA strand breaks, TUNEL assay, Oxidative DNA damage (8-OHdG), FISH for chromosomal translocation

Total: 112

1st Evaluation by specific areas - Keywords to Hepatotoxicity

Liver histology, steatosis, vacuolization, Nile Red, Oil Red O staining, lipid accumulation, Hepatocyte apoptosis, Oxidative stress, GSH, Liver-specific enzymes, ALT, AST, Lipid metabolism markers, Liver morphology imaging

Total: 3,060

1st Evaluation by specific areas - Keywords to Immunotoxicity

Expression of cytokines, IL-1β, TNF-α, IL-6, IL-10, Immune cell infiltration, macrophages, neutrophils, Phagocytic activity assay, NO production, Leukocyte recruitment, NF-κB, ROS quantification, Immune suppression

Total: 2,048

1st Evaluation by specific areas - Keywords to Reproductive toxicity

fertility rate, number of eggs, Egg fertilization, Gonadal histopathology, Oocyte staging, Vitellogenin, estrogenic activity, Sex ratio alteration, Testicular morphology, ovarian morphology, estrogen, progesterone, testosterone

Total: 808

1st Evaluation by specific areas - Keywords to Developmental toxicity

Teratogenicity, Structural malformations, Anomalies, Skeletal variations, Structural variations, Embryo length, Growth retardation, Developmental retardation, Embryo growth, Embryo development, Offspring viability, Body length, Developmental delay, Growth deficits, Eye development, Ocular distance, Eye distance, Hatching Rate, Eclosion Rate, Delayed Hatching, Edema, Pericardial Edema, Yolk Sac Edema, Heart Rate, Blood Flow, blood Circulation, Pigmentation, Notochord Defects, Spontaneous Movement/Swimming Activity, Yolk Consumption, malformation

Total: 3263

Total articles after all filters above: 17,727

Data to generate figure 3:

Classification of zebrafish by age:

This section describes the method used to identify and categorize the developmental stage of zebrafish as used in each reviewed article to observe the embryos, larvae, juveniles, and adults by age.

Keywords to embryo (0 to 3 dpf): Total: 6,178

0 hpf, 1 hpf, 2 hpf, 3 hpf, 4 hpf, 5 hpf, 6 hpf, 7 hpf, 8 hpf, 9 hpf, 10 hpf, 11 hpf, 12 hpf, 13 hpf, 14 hpf, 15 hpf, 16 hpf, 17 hpf, 18 hpf, 19 hpf, 20 hpf, 21 hpf, 22 hpf, 23 hpf, 24 hpf, 25 hpf, 26 hpf, 27 hpf, 28 hpf, 29 hpf, 30 hpf, 31 hpf, 32 hpf, 33 hpf, 34 hpf, 35 hpf, 36 hpf, 37 hpf, 38 hpf, 39 hpf, 40 hpf, 41 hpf, 42 hpf, 43 hpf, 44 hpf, 45 hpf, 46 hpf, 47 hpf, 48 hpf, 49 hpf, 50 hpf, 51 hpf, 52 hpf, 53 hpf, 54 hpf, 55 hpf, 56 hpf, 57 hpf, 58 hpf, 59 hpf, 60 hpf, 61 hpf, 62 hpf, 63 hpf, 64 hpf, 65 hpf, 66 hpf, 67 hpf, 68 hpf, 69 hpf, 70 hpf, 71 hpf, 72 hpf, 1dpf, 2 dpf, 3 dpf, embryo, embryos.

Keywords to larvae (4 to 29 dpf): Total: 5,135

4 dpf, 5 dpf, 6 dpf, 7 dpf, 8 dpf, 9 dpf, 10 dpf, 11 dpf, 12 dpf, 13 dpf, 14 dpf, 15 dpf, 16 dpf, 17 dpf, 18 dpf, 19 dpf, 20 dpf, 21 dpf, 22 dpf, 23 dpf, 24 dpf, 25 dpf, 26 dpf, 27 dpf, 28 dpf, 29 dpf, larvae, larval.

Keywords to juvenile (30 to 89 dpf): Total: 429

30 dpf, 31 dpf, 32 dpf, 33 dpf, 34 dpf, 35 dpf, 36 dpf, 37 dpf, 38 dpf, 39 dpf, 40 dpf, 41 dpf, 42 dpf, 43 dpf, 44 dpf, 45 dpf, 46 dpf, 47 dpf, 48 dpf, 49 dpf, 50 dpf, 51 dpf, 52 dpf, 53 dpf, 54 dpf, 55 dpf, 56 dpf, 57 dpf, 58 dpf, 59 dpf, 60 dpf, 61 dpf, 62 dpf, 63 dpf, 64 dpf, 65 dpf, 66 dpf, 67 dpf, 68 dpf, 69 dpf, 70 dpf, 71 dpf, 72 dpf, 73 dpf, 74 dpf, 75 dpf, 76 dpf, 77 dpf, 78 dpf, 79 dpf, 80 dpf, 81 dpf, 82 dpf, 83 dpf, 84 dpf, 85 dpf, 86 dpf, 87 dpf, 88 dpf, 89 dpf, 90 dpf, 1 mpf, 2 mpf, 3 mpf, juvenile

Keywords to adult (90 dpf to 2 years): Total: 3,229

Adult, adults, 3 mpf, 4 mpf, 5 mpf, 6 mpf, 7 mpf, 8 mpf, 9 mpf, 10 mpf, 11 mpf, 12 mpf, 13 mpf, 14 mpf, 15 mpf, 16 mpf, 17 mpf, 18 mpf, 19 mpf, 20 mpf, 21 mpf, 22 mpf, 23 mpf, 24 mpf.

Next step of the study was evaluate the zebrafish transition between other stages.

The next step of this study is to quantify how many articles progress from the embryonic stage to the larval, juvenile, and adult stages. We will also determine how many studies transition from the larval stage to the juvenile and adult stages, and how many juvenile-stage studies progress to the adult stage. These transitions will be systematically identified and categorized to assess patterns in developmental-stage coverage across the literature.

Embryonic stage to larval stage (0 hpf to 29 dpf): Total 1,803

Keywords used - The article needs to be the at least one keyword to embryo and larvae in the same article:

0 hpf, 1 hpf, 2 hpf, 3 hpf, 4 hpf, 5 hpf, 6 hpf, 7 hpf, 8 hpf, 9 hpf, 10 hpf, 11 hpf, 12 hpf, 13 hpf, 14 hpf, 15 hpf, 16 hpf, 17 hpf, 18 hpf, 19 hpf, 20 hpf, 21 hpf, 22 hpf, 23 hpf, 24 hpf, 25 hpf, 26 hpf, 27 hpf, 28 hpf, 29 hpf, 30 hpf, 31 hpf, 32 hpf, 33 hpf, 34 hpf, 35 hpf, 36 hpf, 37 hpf, 38 hpf, 39 hpf, 40 hpf, 41 hpf, 42 hpf, 43 hpf, 44 hpf, 45 hpf, 46 hpf, 47 hpf, 48 hpf, 49 hpf, 50 hpf, 51 hpf, 52 hpf, 53 hpf, 54 hpf, 55 hpf, 56 hpf, 57 hpf, 58 hpf, 59 hpf, 60 hpf, 61 hpf, 62 hpf, 63 hpf, 64 hpf, 65 hpf, 66 hpf, 67 hpf, 68 hpf, 69 hpf, 70 hpf, 71 hpf, 72 hpf, 1dpf, 2 dpf, 3 dpf, embryo, embryos

+

4 dpf, 5 dpf, 6 dpf, 7 dpf, 8 dpf, 9 dpf, 10 dpf, 11 dpf, 12 dpf, 13 dpf, 14 dpf, 15 dpf, 16 dpf, 17 dpf, 18 dpf, 19 dpf, 20 dpf, 21 dpf, 22 dpf, 23 dpf, 24 dpf, 25 dpf, 26 dpf, 27 dpf, 28 dpf, 29 dpf, larvae, larval

Year	Embryonic stage to larval stage
2014	74
2015	72
2016	103
2017	110
2018	139
2019	178

2020	176
2021	206
2022	237
2023	242
2024	266

Embryonic stage to juvenile (30 dpf to 90 dpf): Total: 119

Keywords used - The article needs to be the at least one keyword to embryo and juvenile in the same article:

0 hpf, 1 hpf, 2 hpf, 3 hpf, 4 hpf, 5 hpf, 6 hpf, 7 hpf, 8 hpf, 9 hpf, 10 hpf, 11 hpf, 12 hpf, 13 hpf, 14 hpf, 15 hpf, 16 hpf, 17 hpf, 18 hpf, 19 hpf, 20 hpf, 21 hpf, 22 hpf, 23 hpf, 24 hpf, 25 hpf, 26 hpf, 27 hpf, 28 hpf, 29 hpf, 30 hpf, 31 hpf, 32 hpf, 33 hpf, 34 hpf, 35 hpf, 36 hpf, 37 hpf, 38 hpf, 39 hpf, 40 hpf, 41 hpf, 42 hpf, 43 hpf, 44 hpf, 45 hpf, 46 hpf, 47 hpf, 48 hpf, 49 hpf, 50 hpf, 51 hpf, 52 hpf, 53 hpf, 54 hpf, 55 hpf, 56 hpf, 57 hpf, 58 hpf, 59 hpf, 60 hpf, 61 hpf, 62 hpf, 63 hpf, 64 hpf, 65 hpf, 66 hpf, 67 hpf, 68 hpf, 69 hpf, 70 hpf, 71 hpf, 72 hpf, 1dpf, 2 dpf, 3 dpf, embryo, embryos

+

30 dpf, 31 dpf, 32 dpf, 33 dpf, 34 dpf, 35 dpf, 36 dpf, 37 dpf, 38 dpf, 39 dpf, 40 dpf, 41 dpf, 42 dpf, 43 dpf, 44 dpf, 45 dpf, 46 dpf, 47 dpf, 48 dpf, 49 dpf, 50 dpf, 51 dpf, 52 dpf, 53 dpf, 54 dpf, 55 dpf, 56 dpf, 57 dpf, 58 dpf, 59 dpf, 60 dpf, 61 dpf, 62 dpf, 63 dpf, 64 dpf, 65 dpf, 66 dpf, 67 dpf, 68 dpf, 69 dpf, 70 dpf, 71 dpf, 72 dpf, 73 dpf, 74 dpf, 75 dpf, 76 dpf, 77 dpf, 78 dpf, 79 dpf, 80 dpf, 81 dpf, 82 dpf, 83 dpf, 84 dpf, 85 dpf, 86 dpf, 87 dpf, 88 dpf, 89 dpf, 90 dpf, 1 mpf, 2 mpf, 3 mpf, juvenile

Year	Embryonic stage to juvenile stage
2014	5
2015	8
2016	7
2017	8
2018	12
2019	17
2020	11
2021	10
2022	19
2023	15

2024	7

Embryonic stage to adult (3 mpf to 2 years): Total: 691

Keywords used - The article needs to be the at least one keyword to embryo and adult in the same article:

0 hpf, 1 hpf, 2 hpf, 3 hpf, 4 hpf, 5 hpf, 6 hpf, 7 hpf, 8 hpf, 9 hpf, 10 hpf, 11 hpf, 12 hpf, 13 hpf, 14 hpf, 15 hpf, 16 hpf, 17 hpf, 18 hpf, 19 hpf, 20 hpf, 21 hpf, 22 hpf, 23 hpf, 24 hpf, 25 hpf, 26 hpf, 27 hpf, 28 hpf, 29 hpf, 30 hpf, 31 hpf, 32 hpf, 33 hpf, 34 hpf, 35 hpf, 36 hpf, 37 hpf, 38 hpf, 39 hpf, 40 hpf, 41 hpf, 42 hpf, 43 hpf, 44 hpf, 45 hpf, 46 hpf, 47 hpf, 48 hpf, 49 hpf, 50 hpf, 51 hpf, 52 hpf, 53 hpf, 54 hpf, 55 hpf, 56 hpf, 57 hpf, 58 hpf, 59 hpf, 60 hpf, 61 hpf, 62 hpf, 63 hpf, 64 hpf, 65 hpf, 66 hpf, 67 hpf, 68 hpf, 69 hpf, 70 hpf, 71 hpf, 72 hpf, 1dpf, 2 dpf, 3 dpf, embryo, embryos

+

Adult, adults, 3 mpf, 4 mpf, 5 mpf, 6 mpf, 7 mpf, 8 mpf, 9 mpf, 10 mpf, 11 mpf, 12 mpf, 13 mpf, 14 mpf, 15 mpf, 16 mpf, 17 mpf, 18 mpf, 19 mpf, 20 mpf, 21 mpf, 22 mpf, 23 mpf, 24 mpf.

Year	Embryonic stage to adult stage
2014	47
2015	42
	61
2017	54
2018	69
2019	54
2020	60
2021	68
2022	63
2023	67
2024	96

Larval stage to juvenile stage (29 hpf to 3 mpf): Total:164

Keywords used - The article needs to be the at least one keyword to larval and juvenile in the same article:

4 dpf, 5 dpf, 6 dpf, 7 dpf, 8 dpf, 9 dpf, 10 dpf, 11 dpf, 12 dpf, 13 dpf, 14 dpf, 15 dpf, 16 dpf, 17 dpf, 18 dpf, 19 dpf, 20 dpf, 21 dpf, 22 dpf, 23 dpf, 24 dpf, 25 dpf, 26 dpf, 27 dpf, 28 dpf, 29 dpf, larvae, larval.

+

30 dpf, 31 dpf, 32 dpf, 33 dpf, 34 dpf, 35 dpf, 36 dpf, 37 dpf, 38 dpf, 39 dpf, 40 dpf, 41 dpf, 42 dpf, 43 dpf, 44 dpf, 45 dpf, 46 dpf, 47 dpf, 48 dpf, 49 dpf, 50 dpf, 51 dpf, 52 dpf, 53 dpf, 54 dpf, 55 dpf, 56 dpf, 57 dpf, 58 dpf, 59 dpf, 60 dpf, 61 dpf, 62 dpf, 63 dpf, 64 dpf, 65 dpf, 66 dpf, 67 dpf, 68 dpf, 69 dpf, 70 dpf, 71 dpf, 72 dpf, 73 dpf, 74 dpf, 75 dpf, 76 dpf, 77 dpf, 78 dpf, 79 dpf, 80 dpf, 81 dpf, 82 dpf, 83 dpf, 84 dpf, 85 dpf, 86 dpf, 87 dpf, 88 dpf, 89 dpf, 90 dpf, 1 mpf, 2 mpf, 3 mpf, juvenile

Year	Larval stage to juvenile stage
2014	5
2015	12
2016	7
2017	8
2018	18
2019	12
2020	20
2021	20
2022	23
2023	23
2024	24

Juvenile stage to adult stage (30 dpf to 3 mpf): Total: 160

Keywords used - The article needs to be the at least one keyword to juvenile and adult in the same article:

30 dpf, 31 dpf, 32 dpf, 33 dpf, 34 dpf, 35 dpf, 36 dpf, 37 dpf, 38 dpf, 39 dpf, 40 dpf, 41 dpf, 42 dpf, 43 dpf, 44 dpf, 45 dpf, 46 dpf, 47 dpf, 48 dpf, 49 dpf, 50 dpf, 51 dpf, 52 dpf, 53 dpf, 54 dpf, 55 dpf, 56 dpf, 57 dpf, 58 dpf, 59 dpf, 60 dpf, 61 dpf, 62 dpf, 63 dpf, 64 dpf, 65 dpf, 66 dpf, 67 dpf, 68 dpf, 69 dpf, 70 dpf, 71 dpf, 72 dpf, 73 dpf, 74 dpf, 75 dpf, 76 dpf, 77 dpf, 78 dpf, 79 dpf, 80 dpf, 81 dpf, 82 dpf, 83 dpf, 84 dpf, 85 dpf, 86 dpf, 87 dpf, 88 dpf, 89 dpf, 90 dpf, 1 mpf, 2 mpf, 3 mpf, juvenile

+

Adult, adults, 3 mpf, 4 mpf, 5 mpf, 6 mpf, 7 mpf, 8 mpf, 9 mpf, 10 mpf, 11 mpf, 12 mpf, 13 mpf, 14 mpf, 15 mpf, 16 mpf, 17 mpf, 18 mpf, 19 mpf, 20 mpf, 21 mpf, 22 mpf, 23 mpf, 24 mpf.

Year	Juvenile stage to adult stage

2014	8
2015	10
2016	8
2017	11
2018	17
2019	18
2020	23
2021	12
2022	21
2023	18
2024	14

Larval stage to adult stage (30 dpf to 3 mpf): Total: 824

Keywords used - The article needs to be the at least one keyword to larval and juvenile in the same article:

4 dpf, 5 dpf, 6 dpf, 7 dpf, 8 dpf, 9 dpf, 10 dpf, 11 dpf, 12 dpf, 13 dpf, 14 dpf, 15 dpf, 16 dpf, 17 dpf, 18 dpf, 19 dpf, 20 dpf, 21 dpf, 22 dpf, 23 dpf, 24 dpf, 25 dpf, 26 dpf, 27 dpf, 28 dpf, 29 dpf, larvae, larval

+

Adult, adults, 3 mpf, 4 mpf, 5 mpf, 6 mpf, 7 mpf, 8 mpf, 9 mpf, 10 mpf, 11 mpf, 12 mpf, 13 mpf, 14 mpf, 15 mpf, 16 mpf, 17 mpf, 18 mpf, 19 mpf, 20 mpf, 21 mpf, 22 mpf, 23 mpf, 24 mpf.

Year	Larval stage to adult stage
2014	42
2015	50
2016	50
2017	61
2018	75
2019	72
2020	77
2021	83

2022	110
2023	93
2024	111

Data to generate figure 4:

Top ten newspapers with the highest number of publications:

Chemosphere: 627 articles

The science of the total environment: 625 articles

Scientific reports: 562 articles

Aquatic toxicology (amsterdam, netherlands): 492 articles

Ecotoxicology and environmental safety: 430 articles

International journal of molecular sciences: 413 articles

Environmental pollution (barking, essex : 1987): 391 articles

Plos one: 360 articles

Fish & shellfish immunology: 300 articles

Comparative biochemistry and physiology. toxicology & pharmacology: 280 articles

Number of articles by year:

	2014	2015	2016	2017	2018	2019	2020	2021	2022	2023	2024
Number of articles	967	1094	1312	1418	1600	1821	2097	2305	2476	2394	2807

Data to generate figure 6:

<u>Classification of Studies by Developmental Stage and Research Area:</u>

To characterize how developmental stages are used across the zebrafish toxicology literature, we categorized each study according to the developmental stage assessed: embryo, larva, juvenile, or adult. For each article, we recorded the primary developmental stage investigated and assigned it to one of the following research areas: neurotoxicity, cardiotoxicity, acute

toxicity, genotoxicity, hepatotoxicity, immunotoxicity, reproductive toxicity, or developmental toxicity. We then grouped all studies by year of publication and quantified the number of articles within each developmental stage for every year. In parallel, we calculated the distribution of developmental stages within each research area, allowing us to compare how different fields prioritize specific stages of the zebrafish life cycle.

<u>Embryo stage by area of study and year:</u>

	2014	2015	2016	2017	2018	2019	2020	2021	2022	2023	2024
Neurotoxicity<br>Total: 2,651	129	138	163	165	187	229	262	317	332	363	366
Cardiotoxicity<br>Total: 1,233	60	59	81	64	94	95	137	148	160	162	173
Acute toxicity<br>Total: 4,223	195	225	280	313	350	402	411	469	504	511	563
Genotoxicity<br>Total: 283	13	17	20	24	21	27	31	28	30	32	40
Hepatotoxicity<br>Total: 980	32	32	49	50	71	85	92	117	144	148	160
Immunotoxicity<br>Total: 750	31	43	46	58	57	70	72	68	86	103	116
Reproductive toxicity	37	37	50	45	47	67	50	46	48	50	56

Total: 533											
Developmental toxicity<br><br>Total: 2,088	120	137	169	173	190	225	270	305	333	340	366

Larval stage by area of study and year:

	2014	2015	2016	2017	2018	2019	2020	2021	2022	2023	2024
Neurotoxicity<br><br>Total: 2,902	101	112	160	182	194	230	300	356	392	401	474
Cardiotoxicity<br><br>Total: 687	23	21	35	33	42	53	72	89	96	96	127
acute toxicity<br><br>Total: 3,118	107	125	179	195	247	284	310	361	432	439	501
Genotoxicity<br><br>Total: 131	4	5	9	12	7	14	13	17	17	16	17
Hepatotoxicity<br><br>Total: 829	21	20	26	40	51	66	80	101	117	139	168

Immunotoxicity<br><br>Total: 836	21	34	31	48	52	71	92	106	113	126	142
Reproductive toxicity<br><br>Total: 374	15	23	24	23	34	51	38	32	41	43	50
Developmental toxicity<br><br>Total: 1,386	36	39	65	82	90	102	143	156	186	223	264

<u>Juvenile stage by area of study and year:</u>

	2014	2015	2016	2017	2018	2019	2020	2021	2022	2023	2024
Neurotoxicity<br><br>Total: 199	7	7	4	10	18	17	28	22	34	23	29
Cardiotoxicity<br><br>Total: 34	1	0	4	0	3	3	3	5	9	5	1
Acute toxicity<br><br>Total: 272	16	20	12	15	23	26	34	22	38	36	30
Genotoxicity<br><br>Total: 34	2	1	4	1	2	4	4	6	7	2	1

Hepatotoxicity<br><br>Total: 52	3	0	1	5	3	6	9	4	5	8	8
Immunotoxicity<br><br>Total: 52	0	1	2	4	4	5	6	6	6	5	13
Reproductive toxicity<br><br>Total: 99	7	7	10	9	13	9	10	8	10	8	8
Developmental toxicity<br><br>Total: 76	4	2	3	7	9	8	8	9	8	10	8

Adult stage by area of study and year:

	2014	2015	2016	2017	2018	2019	2020	2021	2022	2023	2024
Neurotoxicity<br><br>Total: 1,781	70	97	100	130	132	135	194	223	229	224	247
Cardiotoxicity<br><br>Total: 230	14	11	21	14	16	21	24	28	25	24	32
Acute toxicity<br><br>Total: 1,888	91	101	113	129	162	156	199	213	235	221	268

Genotoxicity<br><br>Total: 123	3	3	8	8	19	9	10	14	14	19	16
Hepatotoxicity<br><br>Total: 507	10	19	20	28	40	36	45	62	62	77	108
Immunotoxicity<br><br>Total: 485	12	18	29	37	39	34	53	55	55	66	87
Reproductive toxicity<br><br>Total: 477	16	28	56	37	51	51	45	44	53	32	64
Developmental toxicity<br><br>Total: 444	18	25	37	26	34	33	36	44	51	64	76

Data to generate figure 7:

1nd Inclusion by valuation by application domain - Keywords to Environmental

Water pollution, Industrial effluents, Wastewater, Agricultural runoff, Environmental contaminants, Xenobiotics, Endocrine disruptors, Pesticides, Herbicides, Insecticides, Fungicides, Heavy metals, Trace elements, Microplastics, Nanoplastics, Pharmaceuticals, Personal care products, Antibiotics, Antidepressants, Nanoparticles, Nanomaterials, Persistent organic pollutants (POPs), Polycyclic aromatic hydrocarbons (PAHs), Dioxins, PCBs, Organochlorine compounds, Temperature stress, Salinity, pH changes, UV radiation, Climate change factors, Bioaccumulative substances, Mixture toxicity, Emerging contaminants, Flame retardants, Surfactants, Disinfection by-products, Plastic, microplastic, nanoplastic, Field contaminants, Sediment-bound pollutants, Nutrient pollution (eutrophication), Environmental estrogens, Environmental, oxidative stressors

Glyphosate, Atrazine, Paraquat, Diuron, Metolachlor, Simazine, Alachlor, Acetochlor, Glufosinate, Pendimethalin, Metribuzin, Imazapyr, Imazethapyr, Chlorpyrifos, Malathion, Diazinon, Fipronil, Deltamethrin, Cypermethrin, Permethrin, Imidacloprid,

Thiamethoxam, Acetamiprid, Carbaryl, Aldicarb, Methomyl, Mancozeb, Chlorothalonil, Carbendazim, Azoxystrobin, Tebuconazole, Propiconazole, Trifloxystrobin, Captan, Thiophanate-methyl, Metalaxyl, Ziram, Endosulfan, DDT, Lindane, Rotenone, Copper sulfate, Spinosad, Abamectin, Bifenthrin, Fenvalerate, Trichlorfon, Agrochemical, Pesticide, Herbicide, Insecticide, Fungicide, Acaricide, Biocide, Agricultural runoff, Crop protection, Organophosphate, Organochlorine, Pyrethroid, Carbamate, Neonicotinoid, Agrochemicals, Agricultural chemicals, Rodenticides

Total: 5598

Chemical classification - Keywords for:

Chemical classification Organophosphates,

Chlorpyrifos, Malathion, Diazinon, Trichlorfon, Parathion, Dichlorvos, Organophosphate = 207

Chemical classification Organochlorines,

DDT, Lindane, Endosulfan, PCBs, Organochlorine = 117

Chemical classification Pyrethroids,

Deltamethrin, Cypermethrin, Permethrin, Bifenthrin, Fenvalerate, Pyrethroid = 123

Chemical classification Carbamates,

Carbaryl, Aldicarb, Methomyl, Carbendazim, Carbamate = 65

Chemical classification Neonicotinoids,

Imidacloprid, Thiamethoxam, Acetamiprid, Neonicotinoid = 77

Chemical classification Triazines,

Atrazine, Simazine, Metribuzin = 51

Chemical classification Chloroacetanilides,

Alachlor, Acetochlor, Metolachlor = 29

Chemical classification Ureas, Sulfonylureas,

Diuron = 13

Chemical classification Dinitroanilines,

Pendimethalin = 5

Chemical classification Imidazolines,

Imazapyr, Imazethapyr = 1

Chemical classification Strobilurins,

Azoxystrobin, Trifloxystrobin = 30

Chemical classification Triazoles:

Tebuconazole, Propiconazole = 43

Unclassified compounds:

Glyphosate, Glufosinate, Mancozeb, Chlorothalonil, Captan, Thiophanate-methyl, Metalaxyl, Ziram, Rotenone, Copper sulfate, Spinosad, Abamectin, Agrochemical, Pesticide, Herbicide, Insecticide, Fungicide, Acaricide, Biocide, Agrochemicals, Agricultural chemicals, Crop protection, Agricultural runoff, Rodenticides

Total: 989

2nd Inclusion by valuation by application domain - Keywords to Disease model: Total 3,889

Neurological Keywords

Alzheimer's disease, Parkinson's disease, Amyotrophic lateral sclerosis (ALS), Huntington's disease, Epilepsy, Autism, Attention-deficit/hyperactivity disorder (ADHD), Schizophrenia, Depression, Anxiety, Bipolar disorder, Insomnia, Stroke, Traumatic brain injury, Alzheimer, Parkinson

Total: 1,731

Cardiovascular Keywords

Cardiovascular diseases, Arrhythmias, Myocardial infarction, Heart failure, Hypertension, Atherosclerosis, Cardiomyopathy, Heart regeneration

Total: 449

Metabolic Keywords

Metabolic diseases, Obesity, Type 1 diabetes, Type 2 diabetes, Dyslipidemia, Insulin resistance, Fatty liver disease (NAFLD), Metabolic syndrome

Total: 374

Hepatic Keywords

Liver diseases, Cirrhosis, Liver fibrosis, Drug-induced hepatitis, Cholestatic diseases, Acute liver injury, hepatitis

Total: 103

Renal Keywords

Kidney diseases, Polycystic kidney disease, Acute kidney injury, Glomerulonephritis, Drug-induced nephropathy, Nephrotic syndrome

Total: 53

Respiratory Keywords

Lung diseases, Pulmonary fibrosis, Asthma, Chronic obstructive pulmonary disease (COPD), Hypoxia

Total: 276

Autoimmune Keywords

Autoimmune diseases, Rheumatoid arthritis, Systemic lupus erythematosus (SLE), Multiple sclerosis, Inflammatory bowel disease (Crohn's disease, Ulcerative colitis), Autoimmune type 1 diabetes

Total: 75

Hematological Keywords

Hematological, Anemia, Thalassemia, Abnormal hematopoiesis, Disseminated intravascular coagulation (DIC), Lymphoproliferative disorders

Total: 120

Muscleskeletal **Keywords**

Muscle diseases, Duchenne muscular dystrophy, Myopathies, Rhabdomyolysis, Skeletal diseases, Osteoporosis, Osteoarthritis, Skeletal dysplasia, Bone regeneration, Fractures, Scoliosis

Total: 231

Visual Keywords

Eye diseases, Age-related macular degeneration, Diabetic retinopathy, Cataracts, Glaucoma, Retinal disorders

Total: 76

Hearing Keywords

Hearing disorders, Congenital deafness, Noise-induced hearing loss, Hair cell regeneration

Total: 32

Skin Keywords

Skin diseases, Psoriasis, Atopic dermatitis, Skin cancer, Wound healing, Chronic wounds

Total: 222

Reproductive Keywords

Reproductive disorders, Infertility, Teratogenesis, Hormonal dysfunction, Endocrine disruption

Total: 312

Immunological Keywords

Immune disorders, Immunodeficiencies, Hyperactive immune response, Chronic inflammation

Total: 60

Rare diseases Keywords

Rare genetic diseases, Rett syndrome, Dravet syndrome, Noonan syndrome, Bloom syndrome, Fanconi anemia, Cystic fibrosis, Bardet-Biedl syndrome, Lysosomal storage diseases, Hereditary ataxias

Total: 76

3rd Inclusion by valuation by application domain - Keywords to Preclinical studies

Preclinical, Pre-clinical, Non-clinical, Toxicological testing, Drug screening, Phenotypic screening, Behavioral assay, Bioassay, Mechanistic study, Dose-response, Pharmacological evaluation, Developmental toxicity, Embryotoxicity, Teratogenicity, Safety assessment, Early-phase study, Molecular mechanism, Target validation, Mode of action, Translational research, Disease model, Genetic model, Knockdown model, CRISPR, Pharmacokinetics, Drug efficacy, Drug toxicity, Biomarker discovery, Therapeutic target, Proof-of-concept, High-throughput screening, Small molecule screening

Total: 2,968

Drug screening:

Toxicological testing, Drug screening, Phenotypic screening, Behavioral assay, Bioassay, Mechanistic study, Safety assessment, High-throughput screening, Small molecule screening, Proof-of-concept

Total: 533

Pharmacology screening Keywords:

Dose-response, Pharmacological evaluation, Pharmacokinetics, Drug efficacy, Drug toxicity, Developmental toxicity, Embryotoxicity, Teratogenicity, Molecular mechanism, Mode of action, Target validation, Therapeutic target, Biomarker discovery

Total: 2,018

Data to generate figure 8:

<u>Approach to analyse the Research Context Categorization (RCC):</u>

The RCC was analyzed by mapping each specific toxicity class (e.g., Acute toxicity, Neurotoxicity...) against the three primary application domains (Preclinical, Environmental, and Disease Model). This approach aimed to determine the primary research context in which each toxicity class is most frequently and critically investigated, thereby providing a clear framework for defining the main purpose of the study.

The following keywords were used to obtain the RCC.

Environmental:

Water pollution, Industrial effluents, Wastewater, Agricultural runoff, Environmental contaminants, Xenobiotics, Endocrine disruptors, Pesticides, Herbicides, Insecticides, Fungicides, Heavy metals, Trace elements, Microplastics, Nanoplastics, Pharmaceuticals, Personal care products, Antibiotics, Antidepressants, Nanoparticles, Nanomaterials, Persistent organic pollutants (POPs), Polycyclic aromatic hydrocarbons (PAHs), Dioxins, PCBs, Organochlorine compounds, Temperature stress, Salinity, pH changes, UV radiation, Climate change factors, Bioaccumulative substances, Mixture toxicity, Emerging contaminants, Flame retardants, Surfactants, Disinfection by-products, Plastic, microplastic, nanoplastic, Field contaminants, Sediment-bound

pollutants, Nutrient pollution (eutrophication), Environmental estrogens, Environmental, oxidative stressors, Glyphosate, Atrazine, Paraquat, Diuron, Metolachlor, Simazine, Alachlor, Acetochlor, Glufosinate, Pendimethalin, Metribuzin, Imazapyr, Imazethapyr, Chlorpyrifos, Malathion, Diazinon, Fipronil, Deltamethrin, Cypermethrin, Permethrin, Imidacloprid, Thiamethoxam, Acetamiprid, Carbaryl, Aldicarb, Methomyl, Mancozeb, Chlorothalonil, Carbendazim, Azoxystrobin, Tebuconazole, Propiconazole, Trifloxystrobin, Captan, Thiophanate-methyl, Metalaxyl, Ziram, Endosulfan, DDT, Lindane, Rotenone, Copper sulfate, Spinosad, Abamectin, Bifenthrin, Fenvalerate, Trichlorfon, Agrochemical, Pesticide, Herbicide, Insecticide, Fungicide, Acaricide, Biocide, Agricultural runoff, Crop protection, Organophosphate, Organochlorine, Pyrethroid, Carbamate, Neonicotinoid, Agrochemicals, Agricultural chemicals, Rodenticides

Preclinical:

Preclinical, Pre-clinical, Non-clinical, Toxicological testing, Drug screening, Phenotypic screening, Behavioral assay, Bioassay, Mechanistic study, Dose-response, Pharmacological evaluation, Developmental toxicity, Embryotoxicity, Teratogenicity, Safety assessment, Early-phase study, Molecular mechanism, Target validation, Mode of action, Translational research, Disease model, Genetic model, Knockdown model, CRISPR, Pharmacokinetics, Drug efficacy, Drug toxicity, Biomarker discovery, Therapeutic target, Proof-of-concept, High-throughput screening, Small molecule screening

Disease model:

Alzheimer’s disease, Parkinson’s disease, Amyotrophic lateral sclerosis (ALS), Huntington’s disease, Epilepsy, Autism, Attention-deficit/hyperactivity disorder (ADHD), Schizophrenia, Depression, Anxiety, Bipolar disorder, Insomnia, Stroke, Traumatic brain injury, Alzheimer, Parkinson, Cardiovascular diseases, Arrhythmias, Myocardial infarction, Heart failure, Hypertension, Atherosclerosis, Cardiomyopathy, Heart regeneration, Metabolic diseases, Obesity, Type 1 diabetes, Type 2 diabetes, Dyslipidemia, Insulin resistance, Fatty liver disease (NAFLD), Metabolic syndrome, Liver diseases, Cirrhosis, Liver fibrosis, Drug-induced hepatitis, Cholestatic diseases, Acute liver injury, hepatitis, Kidney diseases, Polycystic kidney disease, Acute kidney injury, Glomerulonephritis, Drug-induced nephropathy, Nephrotic syndrome, Lung diseases, Pulmonary fibrosis, Asthma, Chronic obstructive pulmonary disease (COPD), Hypoxia, Autoimmune diseases, Rheumatoid arthritis, Systemic lupus erythematosus (SLE), Multiple sclerosis, Inflammatory bowel disease (Crohn’s disease, Ulcerative colitis), Autoimmune type 1 diabetes, Hematological, Anemia, Thalassemia, Abnormal hematopoiesis, Disseminated intravascular coagulation (DIC), Lymphoproliferative disorders, Muscle diseases, Duchenne muscular dystrophy, Myopathies, Rhabdomyolysis, Skeletal diseases, Osteoporosis, Osteoarthritis, Skeletal dysplasia, Bone regeneration, Fractures, Scoliosis, Eye diseases, Age-related macular degeneration, Diabetic retinopathy, Cataracts, Glaucoma, Retinal disorders, Hearing disorders, Congenital deafness, Noise-induced hearing loss, Hair cell regeneration, Skin diseases, Psoriasis, Atopic dermatitis, Skin cancer, Wound healing, Chronic wounds, Reproductive disorders, Infertility, Teratogenesis, Hormonal dysfunction, Endocrine disruption, Immune disorders, Immunodeficiencies, Hyperactive immune response, Chronic inflammation, Rare genetic diseases, Rett syndrome, Dravet syndrome, Noonan syndrome, Bloom syndrome, Fanconi anemia, Cystic fibrosis, Bardet-Biedl syndrome, Lysosomal storage diseases, Hereditary ataxias

Neurotoxicity:

Light/Dark preference, Light/Dark test, Novel Tank Test, Locomotor activity, Thigmotaxis, swimming, Visual motor response, Startle response, escape response, Optokinetic response, Anxiety-like behavior, Seizure-like behavior, Optomotor, aversive, Shoaling, social behavior, Aggressive, Acetylcholinesterase (AChE), Dopaminergic neuron, dopamine, Neurotransmitter, GABA, serotonin, Brain oxidative stress, MDA, SOD, CAT, cortisol, Apoptosis, Caspase-3, TUNEL, caspase-8, GFAP expression, Myelin staining, neurotoxicity, neurobehavioral, neuronal damage, dopaminergic, acetylcholinesterase, brain oxidative stress, neurotransmitter, behavior, neurobehavioural, neurotoxic, neurodevelopment, neuroinflammation, neurodegeneration, CNS, neural toxicity, neuroinflammatory, neuronal toxicity

Cardiotoxicity:

Heart rate measurement (bpm), Pericardial area/edema analysis, blood flow, Circulatory defects, Atrium, ventricle, size and contraction, Arrhythmia quantification, Heart morphology, Blood pooling, cardiotoxicity, heart rate, bradycardia, pericardial edema, circulation, arrhythmia, cardiac output, heart beats

Acute toxicity:

Lethality rate, Survival curves, Developmental delay, Coagulation, Hatching delay, Morphological malformations, Loss of Equilibrium (LOE), Immobility, Sedation, Body decoloration, Tail abnormality, Gill dysfunction, Oedema, Hemorrhage, Respiratory Rate, Sublethal effects, Test concentration, Endpoint concentration, NOEC, LOEC, Observation time, Acute Necrosis, pigmentation absence, pigmentation loss, acute toxicity, LC50, mortality, lethality, survival, exposure, acute exposure, OECD 203, OECD 236, FET

Genotoxicity:

Micronucleus test, Chromosome aberration, γH2AX staining, DNA strand breaks, TUNEL assay, Oxidative DNA damage (8-OHdG), FISH for chromosomal translocation, genotoxicity, DNA damage, comet assay, micronucleus, oxidative DNA damage, chromosome aberration

Hepatotoxicity:

Liver histology, steatosis, vacuolization, Nile Red, Oil Red O staining, lipid accumulation, Hepatocyte apoptosis, Oxidative stress, GSH, Liver-specific enzymes, ALT, AST, Lipid metabolism markers, Liver morphology imaging, hepatotoxicity, liver damage, steatosis, hepatocyte vacuolization, oxidative stress, liver enzyme

Immunotoxicity:

Expression of cytokines, IL-1β, TNF-α, IL-6, IL-10, Immune cell infiltration, macrophages, neutrophils, Phagocytic activity assay, NO production, Leukocyte recruitment, NF-κB, ROS quantification, Immune suppression, immunotoxicity, inflammation, cytokine, interleukin, innate immunity, macrophage, neutrophil, immune suppression, immunity

Reproductive Toxicity:

fertility rate, number of eggs, Egg fertilization, Gonadal histopathology, Oocyte staging, Vitellogenin, estrogenic activity, Sex ratio alteration, Testicular morphology, ovarian

morphology, estrogen, progesterone, testosterone, reproductive toxicity, fertility, spawning, fecundity, ovary, testis, sex hormones, endocrine disruption, steroids

Developmental Toxicity:

Teratogenicity, Structural malformations, Anomalies, Skeletal variations, Structural variations, Embryo length, Growth retardation, Developmental retardation, Embryo growth, Embryo development, Offspring viability, Body length, Developmental delay, Growth deficits, Eye development, Ocular distance, Eye distance, Hatching Rate, Eclosion Rate, Delayed Hatching, Edema, Pericardial Edema, Yolk Sac Edema, Heart Rate, Blood Flow, blood Circulation, Pigmentation, Notochord Defects, Spontaneous Movement/Swimming Activity, Yolk Consumption, malformation, developmental toxicity, developmental toxicology, embryo toxicity, embryotoxicity, embryonic toxicity, larval toxicity, early-life stage toxicity, early developmental exposure, toxic effects on development, sublethal developmental effects

Environmental

RCC Neurotoxicity in Environmental:

Total: 2,661

RCC Cardiotoxicity in Environmental:

Total: 695

RCC Acute toxicity in Environmental:

Total: 4,367

RCC Genotoxicity in Environmental:

Total: 289

RCC Hepatotoxicity in Environmental:

Total: 1,270

RCC Immunotoxicity in Environmental:

Total: 627

RCC Reproductive toxicity in Environmental:

Total: 569

RCC Developmental Toxicity in Environmental:

Total: 1,624

Disease Model

RCC Neurotoxicity in Disease model:

Total: 2,177

RCC Cardiotoxicity in Disease model:

Total: 457

RCC Acute toxicity in Disease model:

Total: 1,811

RCC Genotoxicity in Disease model:

Total: 103

RCC Hepatotoxicity in Disease model:

Total: 736

RCC Immunotoxicity in Disease model:

Total: 817

RCC Reproductive toxicity in Disease model:

Total: 366

RCC Developmental Toxicity in Disease model:

Total: 650

Preclinical

RCC - Neurotoxicity in Preclinical:

Total: 1,426

RCC Cardiotoxicity in Preclinical:

Total: 561

RCC Acute toxicity in Preclinical:

Total: 1,852

RCC Genotoxicity in Preclinical:

Total: 147

RCC Hepatotoxicity in Preclinical:

Total: 562

RCC Immunotoxicity in Preclinical:

Total: 405

RCC Reproductive toxicity in Preclinical:

Total: 197

RCC Developmental Toxicity in Preclinical:

Total: 1,547

Further, we analyzed the countries of each article and arrived at the top 10 countries that produced the most articles between 2014 and 2024.

China — 5.778 articles → 40,50%

United States — 3.473 articles → 24,34%

Germany — 787 articles → 5,52%

India — 719 articles → 5,04%

Brazil — 654 articles → 4,58%

South Korea — 646 articles → 4,53%

Japan — 639 articles → 4,48%

Canada — 583 articles → 4,09%

Italy — 495 articles → 3,47%

England — 493 articles → 3,46%
